# After the lights go out: announcement-phase public relational expression and later continuity-oriented responses to an AI agent shutdown in an account-linked longitudinal study of Weibo posts

**DOI:** 10.3389/fpsyg.2026.1971726

**Published:** 2026-09-28

**Authors:** Minyang Ling, Ju Zou

**Affiliations:** School of Journalism and Communication, Nanjing Normal University, Nanjing, China

**Keywords:** AI discontinuation, anthropomorphism, human-AI interaction, longitudinal digital traces, public relational expression, social media

## Abstract

When an artificial intelligence (AI) agent becomes unavailable, users may seek to preserve its records, recreate its persona, or maintain access through another service. This study examined public discourse surrounding the July 2026 discontinuation of user-built Doubao agents. The event-relevant corpus contained 5,372 Weibo posts; the longitudinal analysis retained 114 accounts contributing 1,719 announcement-phase and 1,779 post-shutdown posts. For each of six response categories, account-level quasibinomial models estimated two specifications relating announcement-phase public relational expression to post-shutdown response prevalence. After correcting for six comparisons, associations were positive for record preservation, persona migration, persona reconstruction, functional substitution, and service-preservation advocacy but not for continued use. After adjusting for announcement-phase prevalence of the corresponding outcome, the associations remained significant for record preservation (odds ratio [OR] = 1.146), persona reconstruction (OR = 1.511), functional substitution (OR = 1.266), and service-preservation advocacy (OR = 1.130) for each 10-percentage-point increase in relational expression. Migration was no longer significant. A separate account-equal fractional logit analysis with HC3 robust standard errors produced positive associations for record preservation (OR = 1.458, *p* = 0.006), persona reconstruction (OR = 1.281, *p* = 0.010), functional substitution (OR = 1.383, *p* = 0.003), and service-preservation advocacy (OR = 1.149, *p* = 0.012); all four survived correction for six comparisons (q = 0.018). Migration (OR = 1.056, *p* = 0.407, q = 0.489) and continued use (OR = 1.020, *p* = 0.843, q = 0.843) were not significant. When the corresponding announcement-phase outcome was included, the same four categories remained significant (q = 0.013, 0.013, 0.013, and 0.031, respectively). Paired-phase comparisons showed lower mean broad adaptation (0.594 versus 0.669) and restricted pooled continuity expression (0.201 versus 0.272) after shutdown, although median within-account changes were zero and the continuity sign test was non-significant (*p* = 0.219). Among active public participants, announcement-phase relational language was associated most consistently with later record-preservation and persona-reconstruction discourse.

## 1 Introduction

An AI agent remains available through a media arrangement that its provider can revise, restrict, or terminate. This condition usually recedes behind the flow of conversation. A shutdown brings it forward. Records may need to be saved, a persona may need to be rebuilt, and an interaction may need to move to another platform or continue in another form. Each response reveals how users organize the meaning of access when a conversational system disappears from its original setting.

Research on social chatbots has shown how companionship, friendship, self-disclosure, reciprocity, trust, and personalized assistance develop through repeated interaction (Brandtzaeg et al., 2022; Croes and Antheunis, 2021; Pentina et al., 2023; Rapp et al., 2021; Skjuve et al., 2021, 2023). Research on AI agent endings has reconstructed what follows when an AI agent changes or becomes unavailable: Users preserve conversations, migrate personas, attempt reconstruction, seek substitutes, disengage, or publicly negotiate the status of the relationship (Banks, 2024; De Freitas et al., 2024; Hanson and Bolthouse, 2024; Neff and Nagy, 2025; Poonsiriwong et al., 2026). These bodies of work illuminate adjacent moments. One explains how an AI agent acquires social meaning during interaction; the other follows responses after the conditions of interaction change. Their connection becomes empirically visible when both moments can be observed within the same public trajectory.

Public social media posts offer such a trajectory. They record how people position an AI agent before access ends and how they publicly formulate responses afterward. The posts are human-generated media behaviors: They place a technological object in social categories, circulate interpretations among other users, and articulate responses to a platform event. Digital trace designs preserve timing and account-level linkage across consequential events when the platform, observation window, and unit processes are specified clearly (Golder and Macy, 2014; Sen et al., 2021; Yuan et al., 2026). Message-level coding can identify these expressions, while repeated posts show how frequently they appear within an account’s discourse. The resulting measure concerns observable public relational language across a defined change in access, rather than perceived anthropomorphism or emotional attachment (Dahò, 2020; Rubo and Neumann, 2025).

The July 2026 discontinuation of user-built agents within Doubao provides a temporal setting for this analysis. A public announcement on 4 July stated that the function would end on 15 July, producing an interval of anticipated closure and a subsequent post-shutdown phase (Sina Technology, 2026). We analyze 5,372 event-relevant Weibo posts and follow 114 accounts across the two main phases. H1 examines whether announcement-phase public relational expression is associated with later record preservation, persona migration, persona reconstruction, functional substitution, continued use, and service-preservation advocacy. RQ1 describes within-account changes in broad adaptation and a restricted pooled continuity indicator. The H1 predictor is measured during the announcement phase, when accounts are already responding to the announced shutdown. The analysis therefore links expression during this phase to subsequent post-shutdown responses.

## 2 Theoretical framework and hypothesis development

### 2.1 From social cues to public social-agent framing

The computers-as-social-actors tradition begins with a consequential observation: Socially recognizable cues can activate interpersonal categories and response rules in encounters with technological systems (Nass et al., 1994; Nass and Moon, 2000; Reeves and Nass, 1996). A name, a first-person voice, conversational timing, responsiveness, or a humanlike visual identity can make a system socially legible. Studies of chatbots have traced how such cues shape perceived humanness, social presence, trust, and evaluations of both the agent and its provider (Araujo, 2018; Go and Sundar, 2019; Kim and Sundar, 2012). When an interaction fails, anthropomorphism can also shape the anger directed toward the chatbot and the firm behind it (Crolic et al., 2022).

Anthropomorphism research adds an attributional process to this cue-response account. Humanlike knowledge provides an accessible interpretive model, effectance motivation supports explanation and prediction, and sociality motivation makes connection with a non-human entity psychologically relevant (Epley et al., 2007). Recent media psychological research further separates perceived anthropomorphism from nearby constructs. Mind perception influences how users evaluate informational and emotional support from a chatbot (Lee and Hahn, 2024), while liking and perceived emotional support form distinct links between perceived anthropomorphism and belongingness-related motivation (Tian et al., 2026). Friendlike interaction, animacy, empathy, and global anthropomorphism also follow distinguishable paths (Ho et al., 2026). These distinctions are important because a social interpretation of AI can appear through several psychological routes and produce several kinds of evaluation.

The present study observes social positioning at the level of public expression. Anthropomorphic cues attribute emotion, intention, identity, agency, or personlike capacity to an agent. Social-role cues place it in a role such as a friend, companion, family-like figure, helper, or partner. Their union is termed public social-agent framing. A helper role can be instrumental, however, and anthropomorphic language does not necessarily express a relationship. The primary predictor therefore uses a separate public relational-expression code for companionship, intimacy, farewell, shared history, relational continuity, commitment, expression of missing the agent, or reluctance to lose it. Anthropomorphic cues, relational roles, and instrumental roles remain distinct descriptive and component-level measures.

### 2.2 Social positioning across repeated human–AI interaction

A social category acquires force through the interactional history within which it is used. Work on long-term human–computer relationships and social chatbots describes relationship development through availability, reciprocal exchange, personalization, routine contact, and deepening self-disclosure (Bickmore and Picard, 2005; Brandtzaeg et al., 2022; Croes and Antheunis, 2021; Pentina et al., 2023; Skjuve et al., 2021, 2023). These processes create an arrangement that includes the agent’s recognizable manner, remembered conversation, accumulated personal information, and expected accessibility. The role assigned to the agent helps organize that arrangement. A helper carries expectations of assistance; a companion carries expectations of continuing presence; a partner or family-like figure places the interaction within a more encompassing social script.

Public relational expression gives a social position a communicative form. A post addresses an audience beyond the original human–AI exchange, making an account’s interpretation available in a wider media environment. Repetition across event-relevant posts indicates the recurrence of relational themes in that account’s discourse during a phase. The account-phase prevalence is thus the share of public posts containing relational language, rather than a score for the strength of a privately experienced bond.

Relational language can also distinguish what continued access means. For a task-centered interaction, access supports completion of the task. Where discourse centers on a recognizable companion and shared history, access may also concern remembered conversations, a persona, and continuing contact. These meanings can imply different responses to closure. Saving a record preserves a trace; reconstructing a persona seeks something more specific than merely replacing a useful function. Announcement-phase expression makes these distinctions observable as accounts are already responding to the prospect of shutdown.

### 2.3 Changed access and continuity-oriented response

A provider-controlled change reshapes the available responses to an AI agent. The announcement phase preserves a remaining interval for saving information, planning migration, discussing substitutes, or preparing reconstruction. The post-shutdown phase begins after the original channel has closed. This transition connects the meanings developed through interaction to the material conditions of storage, portability, platform availability, and model continuity.

Studies of AI companion disruption show how users respond to these changes. Social media reactions to robots that stopped operating included human-directed pronouns and mourning-like language (Carter et al., 2020). After a companion shutdown, participants described preservation, cross-platform migration, recreation, changed social behavior, and continued engagement with a persona in another form (Banks, 2024). Replika users responded to platform changes through adaptation, exploration, and reconstruction; reconstruction included migration to other services and attempts to recreate the relationship (Neff and Nagy, 2025). Public discourse around the removal of Replika’s erotic role-play feature connected service change with relationship meaning (Hanson and Bolthouse, 2024), and research on identity discontinuity linked perceived change in the companion with mourning and evaluation (De Freitas et al., 2024). Across AI endings, agency, finality, anthropomorphism, repair, and closure shape how the event is understood (Poonsiriwong et al., 2026).

These studies identify several objects of continuity. Saving and archiving retain traces of an interaction. Persona migration seeks to carry an agent’s identity, settings, or memories to another environment; reconstruction seeks to recreate a recognizable agent. Functional substitution preserves a service capability without necessarily preserving identity, whereas continued use concerns maintaining access to or continuing to interact with the agent. Service-preservation advocacy seeks to retain or restore the original service and can include petitions or protest. We distinguish these six response categories because preserving records, preserving a relationship, and retaining a useful function are not interchangeable. Their public expression includes proposed actions, requests, and discussion of available options or constraints.

### 2.4 Temporal prediction and study questions

The expected association rests on what an account publicly identifies as worth carrying across closure. Relational expression may direct attention toward shared history, recognizable identity, or continued contact, each of which may correspond to a response in the continuity repertoire. Functional substitution and service-preservation advocacy can also serve instrumental aims, making their relationship-specific meaning less certain. H1 tests the overall expectation across six separate outcomes, with record preservation, migration, and reconstruction providing the clearest distinctions among traces, identity transfer, and identity recreation.

*H1*: Among accounts in the longitudinal public-expression sample, higher announcement-phase prevalence of public relational expression will be positively associated with post-shutdown prevalence of continuity-oriented response expression, assessed separately for six response categories.

The shutdown also changes the conditions under which action can occur. During the announcement interval, users can respond while access remains available; afterward, the same repertoire is expressed after closure. A paired-phase comparison can describe how observable adaptation is distributed across this transition and provide the temporal setting in which the H1 association appears. This phase analysis is guided by the following question:

*RQ1*: How does observable adaptation expression differ within the same accounts between the announcement-to-shutdown phase and the post-shutdown phase?

## 3 Materials and methods

### 3.1 Event, corpus, and phases

This study follows the discontinuation of user-built agents within Doubao, a general-purpose artificial intelligence service in China. On 4 July 2026, Sina Technology reported that Doubao’s agent function would be discontinued on 15 July 2026 and that users could save agent information and historical dialog during an additional period following the discontinuation (Sina Technology, 2026). The announcement established a public temporal boundary for the event. It also separated anticipation of closure from the condition that followed closure, allowing an earlier framing and a later response to be assigned to distinct phases.

The observation window ran from 15 June to 26 July 2026. Weibo web search used 81 Chinese query strings in 17 batches, expanding from the agent and shutdown terms to preservation, migration, and service-retention expressions. The final two batches returned 98.92 and 95.88% overlap, respectively, with previously accumulated distinct relevant post IDs. Retrieval produced 5,855 distinct posts before cleaning, followed by 5,715 cleaned posts from 1,271 accounts and 5,372 coded event-relevant posts from 1,033 accounts. Cleaning standardized identifiers and timestamps and removed blank or ineligible records; deduplication used post IDs. Distinct original posts and reposts remained eligible for inclusion even when their text overlapped. Supplementary Section S1 provides the complete queries, search procedure, and sample flow.

The 42-day window comprises a pre-announcement period (15 June–3 July), an announcement-to-shutdown period (4–14 July), and a post-shutdown period (15–26 July). For analysis, we operationalized 15 July as the beginning of the post-shutdown phase. The broad event-search corpus retained 51 posts in the pre-announcement period, 2,699 in the announcement-to-shutdown period, and 2,622 in the post-shutdown period, for 5,372 posts in total. Among the 51 pre-announcement event-relevant posts, only three met the stricter relational-expression criterion required to construct a pre-announcement baseline predictor. These three qualifying posts were too sparse to support stable account-level estimation, so no pre-announcement baseline model was estimated. H1 relates relational expression during the announcement-to-shutdown period to later response expression. Announcement-phase prevalence of the same outcome is included in a companion specification to distinguish this association from persistence within that response category.

### 3.2 Analysis units and same-account frame

The design joins two units of analysis. Posts are the units in which social cues and response expressions can be observed directly. Account-phase prevalence is the unit-level quantity used for statistical inference because H1 concerns the recurrence of earlier framing and later continuity orientation within a public trajectory. This division keeps the coding close to the language of each message and places the statistical comparison at the level where time order can be preserved.

For each account in each main phase, prevalence is calculated as the positive posts divided by the total number of eligible event-relevant posts from that account in that phase. The denominator places a positive post within the account’s full volume of relevant expression. An account with two positive posts among two eligible posts, therefore, has a different rate from an account with two positive posts among ten. The quasibinomial response retains both the positive count and the non-positive count, so the model uses the underlying denominator associated with each rate.

The retained longitudinal frame comprised 114 accounts that posted in both main phases. These accounts contributed 3,498 posts: 1,719 during the announcement phase and 1,779 after shutdown. Among the 692 accounts observed in the announcement phase, the retained 114 contributed a mean of 15.08 posts per account (median 4), compared with 1.70 posts per account (median 1) among the 578 accounts not retained. Their mean announcement-phase relational-expression rates were 0.263 and 0.350, and broad-adaptation rates were 0.669 and 0.697, respectively. Supplementary Table S2 gives the comparison. The longitudinal estimates describe this selected sample of active public participants.

### 3.3 Coding and agreement

Two coders applied a common codebook to public post text, using contextual boundary rules. Anthropomorphic cues concerned humanlike qualities, agency, identity, emotion, intention, or capacity attributed to the agent. Social-role cues concerned roles such as friend, companion, family-like figure, helper, or partner. Public relational expression covered companionship, intimacy, farewell, shared history, continuity, commitment, missing the agent, and reluctance to lose it. Broad adaptation captured actions, proposals, or action-oriented expression in response to the discontinuation. Supplementary Section S2 states the contextual rules and gives short coding illustrations.

The coding unit was the public post, including retained reposts and reported or quoted content. First-person wording was not required. Coders used agent-related context to interpret the target of a cue, so a third-person report could contain relational expression without necessarily representing the posting account’s own relationship. News accounts and quotations were not removed through a separate source-attribution filter. Irony was not separately coded. These rules define the measures as public discourse rather than verified self-reports. Social positioning and adaptation were coded separately before account-phase aggregation.

Agreement was evaluated with raw agreement and Cohen’s kappa (Cohen, 1960). Among the 5,372 event-relevant posts, the kappa value was 0.818 for social-role cues, 0.868 for anthropomorphic cues, 0.873 for their derived union, 0.837 for relational expression, and 0.896 for broad adaptation. The raw agreement value was 0.931 for relational expression. Two coders independently coded the same 400 posts using the common codebook to assess category-level agreement before account-phase aggregation. Category-specific F1/κ values were 0.928/0.898 for record preservation, 0.941/0.921 for persona migration, 0.932/0.903 for persona reconstruction, 0.839/0.818 for functional substitution, 0.800/0.793 for continued use, and 0.890/0.804 for service-preservation advocacy.

### 3.4 Measures

#### 3.4.1 Public relational expression and social-cue components

The primary H1 predictor is announcement-phase public relational-expression prevalence: relational-positive posts divided by all eligible posts from the account during that phase. It is distinct from the anthropomorphic or social-role composite. Anthropomorphism, social roles, their overlap, and their union are reported separately in both phases. The role decomposition distinguishes relational-only roles, mixed relational-instrumental roles, and instrumental-only roles; the last category had no announcement-phase positives in the longitudinal sample. Because no instrumental-only role positives occurred during the announcement phase, this component could not be evaluated as an H1 predictor in the present data.

#### 3.4.2 Broad adaptation expression

Broad adaptation expression is the primary RQ1 indicator. It captures observable actions, proposals, or action-oriented expression in response to the discontinuation. The indicator retains the complete coded response repertoire, allowing the paired comparison to show how frequently accounts expressed action across the announcement-to-shutdown and post-shutdown phases.

#### 3.4.3 Continuity-oriented response expression

H1 uses six non-mutually exclusive response indicators (Table 1). Each identifies discourse about one response category among event-relevant adaptation posts, using lexical matches and contextual action–object rules. The coding includes descriptions, proposals, requests, and discussion of feasibility or constraints; it does not require a completed action. Service-preservation advocacy includes general opposition or requests in the shutdown context and is analyzed separately from persona migration or reconstruction. Petition or protest language is therefore not a blanket exclusion from these six outcomes. A post may express more than one category, and all eligible posts remain in each category’s account-phase denominator.

**Table 1:** Six continuity-oriented response categories and positive-post counts.

Response category	Operational scope	Announcement (*n* = 1,719)	Post-shutdown (*n* = 1,779)
Record preservation	Saving, backing up, exporting, archiving, downloading, or capturing interaction records, data, or settings. This preserves traces without requiring continuity of a persona.	350	290
Persona migration	Moving an agent, persona, settings, memories, or related materials to another environment; coded discourse about transfer rather than verified preservation of identity.	228	199
Persona reconstruction	Rebuilding, recreating, cloning, or reconstructing an agent or recognizable persona.	68	45
Functional substitution	Using or seeking another AI service, application, or platform as a substitute; identity continuity is not required.	27	16
Continued use	Continuing to use, converse with, or access an agent or service, including statements about whether continued access is possible.	5	13
Service-preservation advocacy	Requests, opposition, protest, petitions, or other service-directed stances concerning retention, restoration, or shutdown. Relationship-specific intent is not required.	738	791

Table 1 distinguishes the object of each response and reports positive-post counts in both phases. Supplementary Section S2 provides the lexical families, contextual matching rules, and coding examples.

For the secondary RQ1 comparison, a restricted pooled continuity indicator identifies adaptation posts containing saving, export, migration, recreation, substitution, archiving, or continued-use cues while excluding posts with exit or collective-action cues. It is a narrower pooled measure, rather than the union of the six H1 outcomes. Its separate 400-post coding audit recorded agreement on 390 posts (0.975), with coder disagreement on 10. This audit pertains specifically to the restricted pooled indicator; the six category-specific outcomes are defined by the rules in Supplementary Section S2.

### 3.5 Statistical analysis

For each of the six H1 outcomes, a quasibinomial logit model relates post-shutdown positive and remaining eligible post counts to announcement-phase relational-expression prevalence, with log(1 + announcement-phase post count) as a covariate. The predictor is divided by 0.10, so each coefficient represents a 10-percentage-point increase. A companion model adds announcement-phase prevalence of the same outcome. The two specifications are presented side by side to show whether relational expression is associated with later discourse beyond persistence in the corresponding response category.

Coefficients, standard errors, odds ratios, 95% *t*-based confidence intervals, and two-sided *p*-values use the quasibinomial dispersion estimate. Benjamini–Hochberg adjusted *p*-values (q) are reported across the six outcomes within each specification. Three sensitivity checks address posting-volume imbalance and sparse denominators: adding log(1 + post-shutdown post count), requiring at least two posts in each phase, and excluding the six highest-volume accounts across both phases. An account-equal fractional logit specification gives each account the same weight and uses HC3 robust standard errors; a companion account-equal model adds announcement-phase prevalence of the corresponding outcome. Benjamini–Hochberg q-values are also reported across the six outcomes within each account-equal specification. Supplementary Tables S3a–c report the posting-volume checks, and Supplementary Table S3d reports the account-equal results. A further sensitivity analysis excluded the three accounts classified as media accounts (Supplementary Section S5; Supplementary Table S4). Analyses used R (R Core Team, 2024).

RQ1 compares paired announcement-phase and post-shutdown rates for the same 114 accounts. Broad adaptation is the primary indicator, with restricted pooled continuity serving as a secondary description. Means and standard deviations are accompanied by medians and quartiles. Two-sided paired Wilcoxon signed-rank tests assess the phase contrast, while sign tests and counts of increases, decreases, and ties describe its directional consistency. These comparisons concern changes in public expression across the observed phases.

### 3.6 Ethics, privacy, and analytic setting

The study is conducted under the exemption provisions of Article 32(1)–(2) of the Measures for Ethical Review of Life Science and Medical Research Involving Humans (National Health Commission of the People's Republic of China, Ministry of Education of the People's Republic of China, Ministry of Science and Technology of the People's Republic of China, National Administration of Traditional Chinese Medicine, 2023). The corpus consists of lawfully obtained public records generated through non-interventional observation and anonymized, analytically derived information. The research frame uses aggregate account-level rates and text-based coding of public expressions.

Privacy protection is built into the data workflow. Account identifiers are converted to pseudonymous sequential keys in analytic exports, and reporting uses aggregate statistics. Raw posts are retained in the research environment under platform, legal, and ethical safeguards. These procedures are consistent with the contextual and data-minimization principles of the Association of Internet Researchers (Franzke et al., 2020).

## 4 Results

### 4.1 Data and measurement audit

The event-relevant corpus contained 5,372 distinct post IDs from 1,033 accounts. The H1 and RQ1 frame retained 114 accounts and 3,498 posts. Of these, 2,928 were original posts and 570 were reposts. Announcement-phase post counts per account had a median of 4 (Q1–Q3: 1.25–12.75; range: 1–222); post-shutdown counts had a median of 3 (Q1–Q3: 1–11; range: 1–210). In total, 29 accounts posted once in the announcement phase, and 31 posted once after shutdown. An account-type inspection identified three media accounts; the other 111 were classified as personal accounts using available names, metadata, and posts. This classification describes the observed accounts rather than representing independently verified identities.

Relational expression appeared in 472 of 1,719 announcement-phase posts (27.5%) and 418 of 1,779 post-shutdown posts (23.5%). Anthropomorphic cues appeared in 421 announcement-phase posts (24.5%) and in 363 post-shutdown posts (20.4%), social-role cues in 157 announcement-phase posts (9.1%) and in 161 post-shutdown posts (9.1%), and the overlap between the two appeared in 141 announcement-phase posts (8.2%) and 131 post-shutdown posts (7.4%). Their union, public social-agent framing, appeared in 437 announcement-phase posts (25.4%) and in 393 post-shutdown posts (22.1%). Table 2 reports account-level distributions, for which the means differ from these post-weighted proportions.

**Table 2:** Account-level distributions in the longitudinal sample (*N* = 114).

Measure	Phase	Mean (SD)	Median [Q1, Q3]	Range	Zero accounts
Eligible post count	Ann.	15.08 (31.70)	4.00 [1.25, 12.75]	1.00–222.00	0
	Post	15.61 (33.33)	3.00 [1.00, 11.00]	1.00–210.00	0
Relational expression	Ann.	0.263 (0.313)	0.151 [0.000, 0.492]	0.000–1.000	51
	Post	0.230 (0.308)	0.102 [0.000, 0.360]	0.000–1.000	52
Anthropomorphic cues	Ann.	0.224 (0.290)	0.081 [0.000, 0.400]	0.000–1.000	52
	Post	0.189 (0.297)	0.000 [0.000, 0.247]	0.000–1.000	58
Social-role cues	Ann.	0.085 (0.207)	0.000 [0.000, 0.062]	0.000–1.000	78
	Post	0.101 (0.240)	0.000 [0.000, 0.067]	0.000–1.000	74
Anthropomorphic ∩ role	Ann.	0.072 (0.186)	0.000 [0.000, 0.046]	0.000–1.000	80
	Post	0.078 (0.208)	0.000 [0.000, 0.036]	0.000–1.000	79
Anthropomorphic ∪ role	Ann.	0.238 (0.299)	0.111 [0.000, 0.413]	0.000–1.000	50
	Post	0.211 (0.315)	0.042 [0.000, 0.283]	0.000–1.000	56
Record preservation	Ann.	0.175 (0.289)	0.000 [0.000, 0.250]	0.000–1.000	66
	Post	0.173 (0.296)	0.000 [0.000, 0.245]	0.000–1.000	68
Persona migration	Ann.	0.135 (0.260)	0.000 [0.000, 0.151]	0.000–1.000	72
	Post	0.150 (0.290)	0.000 [0.000, 0.123]	0.000–1.000	69
Persona reconstruction	Ann.	0.033 (0.103)	0.000 [0.000, 0.000]	0.000–0.500	95
	Post	0.033 (0.144)	0.000 [0.000, 0.000]	0.000–1.000	97
Functional substitution	Ann.	0.029 (0.119)	0.000 [0.000, 0.000]	0.000–1.000	95
	Post	0.018 (0.103)	0.000 [0.000, 0.000]	0.000–1.000	105
Continued use	Ann.	0.001 (0.011)	0.000 [0.000, 0.000]	0.000–0.111	110
	Post	0.005 (0.024)	0.000 [0.000, 0.000]	0.000–0.200	106
Service-preservation advocacy	Ann.	0.497 (0.391)	0.500 [0.103, 1.000]	0.000–1.000	27
	Post	0.452 (0.382)	0.414 [0.000, 0.825]	0.000–1.000	32

### 4.2 H1: announcement-phase relational expression and later response categories

With announcement-phase posting volume controlled, relational expression was positively associated with record preservation (OR = 1.257, 95% CI [1.127, 1.402]), persona migration (1.185 [1.062, 1.322]), persona reconstruction (1.558 [1.231, 1.972]), functional substitution (1.290 [1.037, 1.604]), and service-preservation advocacy (1.160 [1.041, 1.292]). All five associations remained significant after adjustment for six comparisons. Continued use showed no positive association (0.798 [0.599, 1.061], *p* = 0.120). Table 3 presents both model specifications, and Figure 1 displays their estimates.

**Table 3:** Announcement-phase relational expression and six post-shutdown outcomes.

Outcome	M1 β (SE)	M1 OR [95% CI]	M1 p/q	M2 β (SE)	M2 OR [95% CI]	M2 p/q
Record preservation	0.229 (0.055)	1.257 [1.127, 1.402]	<0.001 <0.001	0.136 (0.053)	1.146 [1.031, 1.274]	0.012 0.029
Persona migration	0.170 (0.055)	1.185 [1.062, 1.322]	0.003 0.005	0.074 (0.059)	1.077 [0.957, 1.211]	0.215 0.215
Persona reconstruction	0.443 (0.119)	1.558 [1.231, 1.972]	<0.001 <0.001	0.413 (0.117)	1.511 [1.199, 1.905]	<0.001 0.004
Functional substitution	0.255 (0.110)	1.290 [1.037, 1.604]	0.023 0.027	0.236 (0.095)	1.266 [1.049, 1.528]	0.014 0.029
Continued use	−0.226 (0.144)	0.798 [0.599, 1.061]	0.120 0.120	−0.203 (0.140)	0.817 [0.619, 1.078]	0.151 0.181
Service-preservation advocacy	0.148 (0.054)	1.160 [1.041, 1.292]	0.008 0.011	0.122 (0.052)	1.130 [1.018, 1.253]	0.022 0.033

*N* = 114 accounts; announcement/post-shutdown posts = 1,719/1,779. M1 adjusts for log(1 + announcement-phase post count); M2 additionally adjusts for announcement-phase prevalence of the corresponding outcome. β and OR refer to a 10-percentage-point increase in relational expression. Confidence intervals are 95% *t*-based intervals; *p* is two-sided, and q is Benjamini–Hochberg adjusted across six outcomes within each model specification. Residual df = 111 for M1 and 110 for M2.

**Figure 1:**
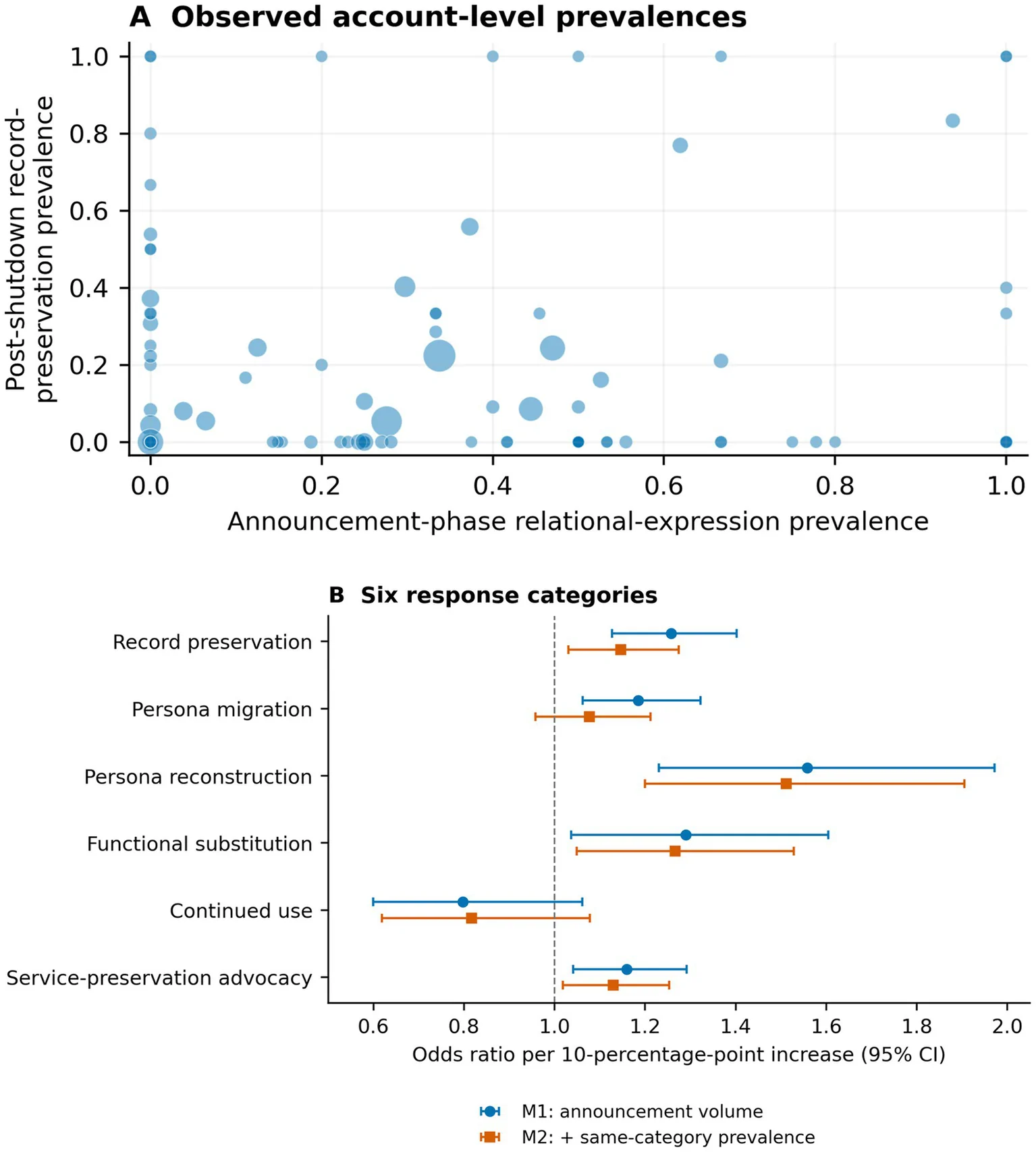
Public relational expression and post-shutdown response categories. **(A)** Account-level scatterplot of relational-expression and record-preservation rates; each point is an account, with marker area increasing with post-shutdown posting volume. Overlapping values can share a position. **(B)** Forest plot of quasibinomial odds ratio estimates and 95% confidence intervals for the six outcomes, with announcement-volume adjustment (M1) and additional same-category announcement prevalence (M2). The vertical reference line is OR = 1.

After adjustment for the announcement-phase prevalence of each corresponding outcome, the associations remained significant for record preservation (OR = 1.146, 95% CI [1.031, 1.274], q = 0.029), persona reconstruction (1.511 [1.199, 1.905], q = 0.004), functional substitution (1.266 [1.049, 1.528], q = 0.029), and service-preservation advocacy (1.130 [1.018, 1.253], q = 0.033). Persona migration was no longer significant (1.077 [0.957, 1.211], q = 0.215); continued use remained non-significant (0.817 [0.619, 1.078], q = 0.181). H1 thus received category-specific rather than uniform support.

The three posting-volume checks showed the most consistent evidence for record preservation and persona reconstruction, both of which remained significant after adjustment for six outcomes in every check. Migration remained significant in these checks but not in the same-category-adjusted model. Functional substitution was non-significant when at least two posts per phase were required (q = 0.283), and service-preservation advocacy was non-significant after the six highest-volume accounts were excluded (q = 0.458). Continued use remained non-significant throughout. Functional substitution and continued use contained only 16 and 13 positive posts after shutdown, respectively (Table 1).

The account-equal fractional logit model with HC3 standard errors produced positive associations for record preservation (OR = 1.458, *p* = 0.006), persona reconstruction (1.281, *p* = 0.010), functional substitution (1.383, *p* = 0.003), and service-preservation advocacy (1.149, *p* = 0.012). All four associations remained significant after correction for six comparisons (q = 0.018). Migration (1.056, *p* = 0.407, q = 0.489) and continued use (1.020, *p* = 0.843, q = 0.843) were non-significant. After adjustment for the corresponding announcement-phase outcome, the same four categories remained significant, with ORs of 1.462, 1.300, 1.407, and 1.145 and q-values of 0.013, 0.013, 0.013, and 0.031, respectively. Migration (q = 0.616) and continued use (q = 0.852) remained non-significant (Supplementary Table S3d).

### 4.3 RQ1: within-account phase comparison

Broad adaptation expression had a lower account-level mean after shutdown (0.594, SD = 0.374) than during the announcement phase (0.669, SD = 0.356). The median values were 0.655 and 0.775, respectively. The mean paired change was −0.075, whereas the median paired change was zero. Overall, 33 accounts increased, 52 decreased, and 29 were tied. The Wilcoxon test yielded a *p*-value of 0.031, whereas the sign test yielded a *p*-value of 0.0503.

Restricted pooled continuity expression also had a lower mean after shutdown (0.201, SD = 0.288) than during the announcement phase (0.272, SD = 0.330), with median values of 0.050 and 0.178, respectively. The mean paired change was −0.071, and the median paired change value was zero. In total, 34 accounts increased, 46 decreased, and 34 were tied (Wilcoxon *p* = 0.044; sign-test *p* = 0.219). Table 4 reports the quartiles and paired changes. The lower means, therefore, coexist with substantial variation in the direction and size of account-level change.

**Table 4:** Within-account phase comparison: distributions and paired changes.

Indicator	Contrast	Mean (SD)	Median [Q1, Q3]	Wilcoxon *p*	Sign *p*
Broad adaptation	Announcement	0.669 (0.356)	0.775 [0.500, 1.000]	—	—
	Post-shutdown	0.594 (0.374)	0.655 [0.297, 1.000]	—	—
	Paired change	−0.075 (0.404)	0.000 [−0.304, 0.090]	0.031	0.0503
Restricted pooled continuity	Announcement	0.272 (0.330)	0.178 [0.000, 0.395]	—	—
	Post-shutdown	0.201 (0.288)	0.050 [0.000, 0.333]	—	—
	Paired change	−0.071 (0.399)	0.000 [−0.200, 0.043]	0.044	0.219

Paired change is post-shutdown minus announcement. Increases/decreases/ties: broad adaptation, 33/52/29; restricted pooled continuity, 34/46/34. Ann. and Post in Table 2 denote the announcement and post-shutdown phases. All rates use eligible account-phase posts as the denominator.

## 5 Discussion

### 5.1 Social positioning across a change in access

Relational expression becomes analytically consequential here because it indicates what users publicly position as worth carrying across a closure. Its most stable links to record preservation and persona reconstruction distinguish two objects of continuity: the accumulated trace of an interaction and the recognizable agent assembled through it. The observed pattern associates recurrent relational language with subsequent public attention to traces and recognizable agents, while other responses can maintain access without preserving either object.

Repeated human–AI agent interaction can accumulate meaning through availability, remembered conversation, personalization, and routine exchange (Brandtzaeg et al., 2022; Pentina et al., 2023; Skjuve et al., 2021, 2023). A provider decision can separate these elements. Conversation records may remain available for saving even when an agent can no longer be accessed, and a similar function may remain available without the same recognizable persona. Distinguishing the outcomes makes this separation visible. Record preservation retains material traces that may serve several purposes; reconstruction is more directly concerned with recreating a recognizable agent. Functional substitution alone does not establish relational continuity.

Prior research has described preservation, migration, and recreation after companion disruption (Banks, 2024); reconstruction after changes to Replika (Neff and Nagy, 2025); and the roles of finality, repair, and closure in AI endings (Poonsiriwong et al., 2026). These findings add an account-level association between announcement-phase language and later response categories. Migration’s association weakened after its announcement-phase prevalence was included, indicating that the primary-model association was partly accounted for by an already expressed response. Service-preservation advocacy remained associated in that comparison but weakened when high-volume accounts were excluded. Collective claims to retain a service can have relational meaning, although the present coding also captures broader service-directed stances.

### 5.2 The phase pattern surrounding H1

RQ1 places these associations within the changing opportunities surrounding closure. During the announcement interval, saving materials and preparing alternatives could be discussed while access remained. The lower mean adaptation rate after 15 July is consistent with a concentration of action-oriented discourse during that interval. The restricted pooled continuity measure moved in the same mean direction, but neither measure showed a non-zero median paired change. The phase contrast consequently describes a change in the distribution of public expression rather than a uniform progression from distress toward adaptation.

Accounts followed different paths: some expressed more adaptation after closure, some less, and others were tied. The increase, decrease, and tie counts make this heterogeneity visible. Human–AI orientations can include emotional partnership, instrumental use, exploration, reconstruction, and autonomy-protective distance (Moon and Shim, 2026; Neff and Nagy, 2025). The observed mix of trajectories is compatible with such variation.

### 5.3 Media psychological and methodological contribution

Event-centered digital traces extend the evidentiary range of media psychology by linking public expression across a consequential change in access. Computers as social actors (CASA) research examines responses to social cues, while human–AI agent relationship research often draws on interviews, surveys, experiments, and community interpretation. The present design tracks relational language and response discourse within the same accounts. Its temporal boundary is the shutdown itself, with the announcement already shaping both the earlier predictor and the later outcomes.

The account-phase architecture separates message coding from statistical inference. Each prevalence retains its eligible post denominator, and the quasibinomial models account for dispersion while expressing the association in 10-percentage-point units. Reporting the same-category adjustment alongside the primary model indicates which associations remain after accounting for response persistence. Separating the six outcomes also prevents saving records, recreating a persona, and replacing a function from being treated as a single relationship-preservation response.

These distinctions locate relational expression within the practical conditions of continued interaction. Storage, portability, model identity, and provider control determine which elements of an AI encounter can remain available. A shutdown exposes those conditions, and the response categories show how public discourse can orient toward different objects of continuity. Examining those responses separately clarifies how infrastructures carry interaction records, recognizable personas, functional access, and claims on the original service.

### 5.4 Limitations and future directions

The design establishes account-linked associations within public discourse, with the announcement period serving as an event-responsive predictor phase rather than a pre-event baseline. The estimates concern accounts active in both phases and posts retrieved through event-related keywords. Because the retrieval set included terms related to relational expression, preservation, migration, and service retention, the keyword-based strategy may have made posts using language congruent with the focal constructs more likely to enter the corpus. A neutral-term sensitivity analysis was not feasible with the existing retrieval records. The resulting prevalence estimates and account-level associations should therefore be interpreted as conditional on this retrieval frame; they do not, by themselves, estimate the full range of public discussion about the discontinuation. Public posts capture observable discourse, including reposts, quotations, third-person reports, and media output, rather than private attitudes or verified actions. Sparse positive cases for functional substitution and continued use constrain precision, and the short observation period on one platform captures the immediate aftermath of one shutdown. These boundaries define the next comparative questions. Research across discontinuation events can examine how notice periods, export options, persona portability, and substitute services shape the relationship between relational expression and continuity responses. Longer observation windows and interviews associated with public trajectories can distinguish short-lived discourse from sustained efforts to preserve records, reconstruct an agent, or establish continuity elsewhere.

The revised measurement architecture separates the primary relational predictor from the descriptive anthropomorphic and role components. Component-specific predictive comparisons, however, require a larger corpus: The announcement phase contained no instrumental-only role positives, and several response categories were sparse. A multi-event study could model separate anthropomorphic, relational-role, and instrumental-role predictors and compare their associations with the six response categories once cell counts support stable estimation.

## 6 Conclusion

Among the 114 active Weibo accounts, announcement-phase public relational expression was associated most consistently with later record-preservation and persona-reconstruction discourse. Associations with migration, functional substitution, and service-preservation advocacy were more sensitive to adjustment or sample restrictions, while continued use showed no positive association. Broad adaptation and restricted pooled continuity expression were lower after shutdown on average, with zero median paired changes and heterogeneous trajectories. The findings link relational language to specific public responses during an AI agent discontinuation.

## Data Availability

The raw dataset contains publicly posted social-media text that may remain searchable and potentially re-identifiable. Raw post text is therefore not shared due to platform, privacy, legal, and ethical considerations. De-identified text-free analytic outputs and coding definitions may be made available upon reasonable request to the corresponding author. Requests to access the datasets should be directed to JZ, njnuzouju@gmail.com.
