# Supplementary material for: After the lights go out: announcement-phase public relational expression and later continuity-oriented responses to an AI agent shutdown in an account-linked longitudinal study of Weibo posts
Source: Front Psychol. 2026 Sep 28;17:1971726. doi: 10.3389/fpsyg.2026.1971726 (PMC13655048; doi:10.3389/fpsyg.2026.1971726)
Supplement: Supplementary file 1 [file Supplementary_file_1.docx]

# 1 Supplementary Material

## 1.1 S1. Retrieval and sample construction

Searches were executed on Weibo's web search interface using date-filtered keyword queries and pagination. The main search interval was 15 June 2026 00:00 to 27 July 2026 00:00, covering posts through 26 July; initial daily slices were also used. The search URL used the q, timescope, and page parameters, without an additional sorting parameter. A representative parameter form is timescope=custom:2026-06-15-0:2026-07-27-0. The broad event-search corpus retained 51 event-relevant posts in the pre-announcement period (15 June–3 July), of which only three met the stricter relational-expression criterion required to construct a pre-announcement baseline predictor. These three qualifying posts were too sparse to support stable account-level estimation, so no pre-announcement baseline model was estimated.

Expansion proceeded through 17 batches using event synonyms, preservation and migration vocabulary, and service-retention expressions. For each batch, overlap was the proportion of its distinct relevant post IDs already present in the accumulated results. The last two batches had 1,387 and 704 relevant IDs, including 15 and 29 newly identified posts, respectively (overlap 98.92% and 95.88%). All 81 query strings below returned hits. This stopping rule describes retrieval saturation under these queries.

Deduplication was by Weibo post ID, so reposts with their own IDs remained distinct public records. The longitudinal frame contained 1,472 original posts and 247 reposts in the announcement phase, and 1,456 original posts and 323 reposts after shutdown. Repeated text across distinct IDs was retained. Account identifiers linked the two phases. Comment text was not used as an outcome or predictor in this manuscript.

Table S1. Retrieval-to-analysis sample flow.

| **Stage** | **N** | **Definition** |
| --- | --- | --- |
| Distinct retrieved posts before cleaning | 5,855 | After query-level overlap was resolved by post ID |
| Cleaned posts | 5,715 | 1,271 accounts |
| Event-relevant posts | 5,372 | 1,033 accounts |
| Pre-announcement / announcement / post-shutdown | 51 / 2,699 / 2,622 | Broad event-search corpus counts; only three pre-announcement posts met the strict baseline-predictor criterion. |
| Longitudinal account sample | 114 | Accounts with posts in both main phases. |
| Longitudinal announcement / post-shutdown posts | 1,719 / 1,779 | Total 3,498; same sample for H1 and RQ1 |

### 1.1.1 Complete query strings (81; spaces retained)

1. 豆包智能体; 2. 豆包智能体下线; 3. 豆包智能体下架; 4. 豆包智能体清退; 5. 豆包智能体删除; 6. 豆包智能体告别; 7. 豆包智能体迁移; 8. 豆包转猫箱; 9. 豆包 拟人化互动管理办法; 10. 拟人化互动管理办法.

11. 反对豆包智能体一刀切; 12. 豆包用户质疑智能体被下架; 13. 豆包千问下架智能体让用户崩溃; 14. 智能体下线与责任之争; 15. 豆包下架智能体隐私风险谁负责; 16. 豆包下架智能体是保护还是背刺; 17. 豆包转移猫箱; 18. 豆包迁移猫箱; 19. 豆包智能体转移猫箱; 20. 豆包智能体迁移猫箱.

21. 豆包智能体 数字亲人 下线; 22. 豆包智能体 情感陪伴 下架; 23. 豆包智能体 精神寄托 消失; 24. 豆包智能体 告别 关系; 25. 豆包智能体 数字资产 下架; 26. 豆包智能体 用户权益 下线; 27. 豆包智能体 完整导出 删除; 28. 豆包智能体 数据清空 迁移; 29. 豆包智能体 一刀切 监管; 30. 豆包智能体 平台责任 下线.

31. 豆包智能体 成年实名 分级管控; 32. 豆包智能体 维权 恢复; 33. 豆包智能体功能将下线; 34. 豆包智能体下线后无法查看数据; 35. 豆包 一键删除用户作品 智能体; 36. 豆包 强制下架 智能体 隐私; 37. 豆包 智能体流产 平台不负责; 38. 豆包 反对智能体下线删除; 39. 豆包 抵制智能体下线; 40. 豆包 恢复智能体功能.

41. 豆包 智能体重新上线; 42. 豆包智能体 AI亲人 下线; 43. 豆包智能体 爱人 消失; 44. 豆包智能体 陪伴 失去; 45. 豆包智能体 关系断裂; 46. 豆包智能体 聊天记录 导出 下线; 47. 豆包智能体 人设记忆 迁移; 48. 豆包智能体 网络虚拟财产 下架; 49. 豆包智能体 字节霸王条款 下线; 50. 豆包千问建议提前备份重要内容.

51. 智能体将下线网友开始集体搬家 豆包; 52. AI数字亲人下线平台推一键导出 豆包; 53. 字节旗下豆包侵吞用户数据资产; 54. 豆包智能体临终遗言大赏; 55. 数字资产不容侵犯 豆包智能体下架; 56. 字节跳动背刺用户 豆包智能体; 57. 豆包就是这样糊弄人的 智能体; 58. 豆包就是这样糊弄人的; 59. 字节跳动背刺用户 豆包下架; 60. 拒绝豆包智能体功能下线.

61. 抵制豆包智能体下架; 62. 反对豆包智能体下架; 63. 反对豆包智能体一刀切下架; 64. 豆包智能体一刀切下架; 65. 还我豆包智能体; 66. 恢复豆包智能体; 67. 取消下架豆包智能体; 68. 豆包还我智能体; 69. 还我智能体 豆包下架; 70. 豆包智能体不要下架.

71. 豆包不要删除智能体; 72. 豆包保留智能体; 73. 不要下架豆包智能体; 74. 不要删除豆包智能体; 75. 保留豆包智能体; 76. 豆包智能体留下来; 77. 豆包智能体别下架; 78. 豆包智能体不要走; 79. 豆包智能体别删除; 80. 豆包智能体继续保留.

81. 豆包智能体恢复上线.

## 1.2 S2. Coding definitions and contextual decisions

Event relevance requires a clear connection to the Doubao-agent shutdown, loss of access, or related event. Anthropomorphic coding identifies attributed humanlike psychology, emotion, identity, memory, or agency. Social-role coding requires the role and an agent or relational cue in the same local semantic unit. Relational expression covers companionship, intimacy, farewell, shared history, continuity, commitment, missing the agent, or reluctance to lose it. Broad adaptation includes preservation, migration, reconstruction, substitution or continued use, collective action, and exit orientation.

Both coder outputs used the common codebook. The contextual rules addressed anthropomorphic targets, relational neighborhoods, social-role cues, relational expressions, and the distinction between actions or proposals in the post body and slogan-only hashtags. Implicit agent references could qualify when the context identified the target. A first-person pronoun was not an inclusion requirement.

The six response indicators require event relevance and a positive adaptation label. Their lexical families are listed below; the contextual branch matches action and object terms in either order, within a limited local context. Advocacy matches service-directed stances and retention or shutdown language in local combinations; general stance matches remain eligible within event-related adaptation discourse. Lexical and contextual matches form each category's positive set. Categories can overlap.

Contextual matches do not impose a universal first-person, completed-action, or negation filter. Accordingly, discussion of an unavailable option can still identify the response being discussed, and a positive label is not evidence that the account performed that action. Original posts, reposts, reported speech, and third-person descriptions use the same post-level rules; irony and source attribution are not separate variables. The following short expressions illustrate coding vocabulary, not quotations from research participants.

Coding illustrations (lexical examples, not participant quotations).

| **Code** | **Chinese illustration** | **English gloss / scope** |
| --- | --- | --- |
| Relational expression | 陪伴; 想念; 不舍 | Companionship; missing; reluctance to lose. |
| Anthropomorphic attribution | 记忆; 情绪; 意图 | Memory, emotion, or intention attributed to the agent in context. |
| Relational / instrumental role | 朋友; 伴侣 / 助手; 老师 | Friend; partner / helper; teacher. Role context determines the category. |
| Record preservation | 保存聊天记录; 导出数据 | Save chat records; export data. |
| Persona migration | 迁移人设; 导入记忆 | Migrate persona settings; import memories. |
| Persona reconstruction | 重建智能体; 复刻角色 | Rebuild the agent; recreate a character. |
| Functional substitution | 改用其他AI; 平替 | Switch to another AI; substitute. |
| Continued use | 继续使用; 还能用 | Continue using; still usable. |
| Service-preservation advocacy | 保留智能体; 反对下架 | Retain the agent; oppose shutdown. |
| Exit in broad adaptation | 退出; 卸载 | Exit; uninstall. Included in broad adaptation, distinct from continuity categories. |

Record preservation: 保存、备份、导出、存档、归档、留存、打包、保留+对象、下载+对象、数据带走、记录带走、截图、录屏.

Persona migration: 迁移、移植、搬到、转到+平台、换平台、导入+对象、转移+对象、上传+对象、复制.

Persona reconstruction: 重建、重构、复刻、克隆、还原+对象、重做、再做一个、重新创建/建立/搭建、新建+对象.

Functional substitution: 平替、换成+对象、换到+对象、改用、转用、去用+对象、用其他AI、转向其他AI、另一个.

Continued use: 继续使用、继续用、接着用、还在用、还能用、仍然使用/用、用户+继续使用.

Service-preservation advocacy: 反对、抵制、抗议、请愿、联名、投诉、呼吁、要求、建议、希望、恳请、请求、拒绝、不要/别.

Contextual object families include interaction records, dialogue, data, history, memory, persona settings, and content for preservation; agents, characters, settings, memories, voices, AI, assistants, and models for migration or reconstruction; and AI services, agents, assistants, platforms, software, applications, or models for substitution and continued use. Service-advocacy patterns combine service or shutdown targets with requests, refusal, opposition, retention, restoration, or reopening language.

## 1.3 S3. Sample composition and posting-volume checks

Table S2. Announcement-phase characteristics of retained and non-retained accounts.

| **Measure** | **Retained mean** | **Retained median** | **Not retained mean** | **Not retained median** |
| --- | --- | --- | --- | --- |
| Announcement post count | 15.079 | 4.000 | 1.696 | 1.000 |
| Anthropomorphic-cue rate | 0.224 | 0.081 | 0.251 | 0.000 |
| Relational-role rate (including mixed) | 0.085 | 0.000 | 0.130 | 0.000 |
| Instrumental-role rate (including mixed) | 0.021 | 0.000 | 0.064 | 0.000 |
| Relational-expression rate | 0.263 | 0.151 | 0.350 | 0.000 |
| Broad-adaptation rate | 0.669 | 0.775 | 0.697 | 1.000 |

Note. The comparison includes all 692 accounts observed in the announcement phase: 114 retained and 578 not retained. It does not compare the 114 accounts against all corpus accounts, some of which were not observed in that phase. Role measures here include mixed relational–instrumental cases in both corresponding rates.

The same-category-adjusted specification in Table 3 addresses persistence in each response. The three additional checks in Tables S3a–S3c address distinct posting-volume risks: phase-specific activity, unstable proportions from single-post phases, and influence of the most prolific accounts. Each check modifies M1, not M2. The two-phase volume model uses all 114 accounts; the minimum-two-post model uses 63; excluding the six largest combined phase totals leaves 108. q values are adjusted across the six outcomes within each specification. The account-equal fractional-logit analysis in Table S3d uses HC3 robust standard errors and reports raw two-sided p values and Benjamini–Hochberg q values. Four outcomes remain significant after correction in the primary account-equal model: record preservation, persona reconstruction, functional substitution, and service-preservation advocacy (all q = .018). The same four remain significant after the corresponding announcement-phase outcome is added (q = .013, .013, .013, and .031, respectively); migration and continued use remain nonsignificant.

Table S3a. Both-phase volume.

| **Outcome** | **Accounts** | **OR [95% CI]** | **p** | **q** |
| --- | --- | --- | --- | --- |
| Record preservation | 114 | 1.251 [1.121, 1.395] | <.001 | <.001 |
| Persona migration | 114 | 1.180 [1.057, 1.319] | .004 | .007 |
| Persona reconstruction | 114 | 1.508 [1.213, 1.876] | <.001 | <.001 |
| Functional substitution | 114 | 1.255 [1.011, 1.558] | .040 | .048 |
| Continued use | 114 | 0.796 [0.595, 1.065] | .124 | .124 |
| Service-preservation advocacy | 114 | 1.158 [1.038, 1.291] | .009 | .013 |

Table S3b. At least two posts/phase.

| **Outcome** | **Accounts** | **OR [95% CI]** | **p** | **q** |
| --- | --- | --- | --- | --- |
| Record preservation | 63 | 1.318 [1.131, 1.534] | <.001 | .004 |
| Persona migration | 63 | 1.237 [1.063, 1.440] | .007 | .020 |
| Persona reconstruction | 63 | 1.482 [1.040, 2.112] | .030 | .045 |
| Functional substitution | 63 | 1.201 [0.856, 1.686] | .283 | .283 |
| Continued use | 63 | 0.799 [0.561, 1.139] | .211 | .253 |
| Service-preservation advocacy | 63 | 1.211 [1.036, 1.415] | .017 | .034 |

Table S3c. Exclude top six accounts.

| **Outcome** | **Accounts** | **OR [95% CI]** | **p** | **q** |
| --- | --- | --- | --- | --- |
| Record preservation | 108 | 1.226 [1.105, 1.362] | <.001 | <.001 |
| Persona migration | 108 | 1.168 [1.054, 1.293] | .003 | .007 |
| Persona reconstruction | 108 | 1.324 [1.169, 1.500] | <.001 | <.001 |
| Functional substitution | 108 | 1.267 [1.041, 1.543] | .019 | .028 |
| Continued use | 108 | 0.789 [0.577, 1.079] | .137 | .164 |
| Service-preservation advocacy | 108 | 1.033 [0.948, 1.125] | .458 | .458 |

Table S3d. Account-equal fractional-logit models with HC3 standard errors.

| **Outcome** | **Primary β [95% CI]** | **Primary OR** | p / q | **Adjusted β [95% CI]** | **Adjusted OR** | p / q |
| --- | --- | --- | --- | --- | --- | --- |
| Record preservation | 0.377 [0.106, 0.649] | 1.458 | .006 .018 | 0.380 [0.108, 0.652] | 1.462 | .006 .013 |
| Persona migration | 0.055 [-0.075, 0.185] | 1.056 | .407 .489 | 0.050 [-0.100, 0.200] | 1.051 | .513 .616 |
| Persona reconstruction | 0.247 [0.058, 0.437] | 1.281 | .010 .018 | 0.262 [0.074, 0.451] | 1.300 | .006 .013 |
| Functional substitution | 0.324 [0.109, 0.539] | 1.383 | .003 .018 | 0.341 [0.106, 0.577] | 1.407 | .004 .013 |
| Continued use | 0.020 [-0.179, 0.220] | 1.020 | .843 .843 | 0.019 [-0.179, 0.216] | 1.019 | .852 .852 |
| Service-preservation advocacy | 0.139 [0.030, 0.247] | 1.149 | .012 .018 | 0.135 [0.021, 0.250] | 1.145 | .021 .031 |

Note. Each account contributes one account-phase rate. The primary model includes the announcement-phase posting-volume covariate; the adjusted model additionally includes announcement-phase prevalence of the corresponding outcome. β and confidence intervals are on the fractional-logit scale, odds ratios correspond to a 10-percentage-point increase in relational expression, p values are raw two-sided HC3 tests, and q values are Benjamini–Hochberg adjusted across the six outcomes within each specification.

## 1.4 S4. Social-cue components and account types

Social-role decomposition distinguishes 124 announcement-phase posts with relational-only roles, 30 with mixed relational–instrumental roles, and 3 other role-coded posts; there were no instrumental-only posts. After shutdown there were 132 relational-only, 22 mixed, 5 instrumental-only, and 2 other role-coded posts. An instrumental-only role component could not be evaluated as a predictor because the announcement phase contained no positive cases.

Account-type inspection identified three media accounts and classified the other 111 as personal using available account information and posts.

## 1.5 S5. Media-account exclusion sensitivity

To assess whether the six-category associations were driven by media accounts, we removed the three media accounts and re-estimated the two specifications on the remaining 111 accounts. M1 retains the announcement-phase posting-volume covariate; M2 additionally controls announcement-phase same-category outcome rate. Supplementary Table S4 reports odds ratios per 10-percentage-point increase in announcement-phase relational expression, with q values adjusted across the six outcomes within each specification.

Table S4. Six-category results after excluding three media accounts.

| **Outcome** | **Post positive posts** | **M1: no same-category control OR [95% CI]** | **M1 p / q** | **M2: announcement-phase same-category control OR [95% CI]** | **M2 p / q** |
| --- | --- | --- | --- | --- | --- |
| Record preservation | 288 | 1.254 [1.122, 1.400] | <.001 <.001 | 1.131 [1.015, 1.261] | .027 .041 |
| Persona migration | 197 | 1.177 [1.053, 1.317] | .005 .008 | 1.062 [0.941, 1.198] | .330 .330 |
| Persona reconstruction | 42 | 1.485 [1.146, 1.924] | .003 .008 | 1.409 [1.095, 1.814] | .009 .029 |
| Functional substitution | 14 | 1.385 [1.096, 1.751] | .008 .009 | 1.378 [1.086, 1.748] | .010 .029 |
| Continued use | 13 | 0.798 [0.599, 1.064] | .127 .127 | 0.817 [0.618, 1.080] | .159 .191 |
| Service-preservation advocacy | 791 | 1.174 [1.052, 1.310] | .005 .008 | 1.142 [1.027, 1.269] | .015 .031 |

Note. The analysis includes 111 accounts after excluding three media accounts. The post-shutdown denominator is 1,775 posts. Odds ratios represent 10-percentage-point increases in announcement-phase relational expression; p values are two-sided and q values are Benjamini–Hochberg adjusted across six outcomes within each specification.
